# Estimates of avoided costs attributed to ashort cervix screening program to preventpreterm birth from the perspective of theUnified Health System (SUS)

**DOI:** 10.11606/s1518-8787.2023057004376

**Published:** 2023-10-30

**Authors:** Thais V. Silva, Anderson Borovac-Pinheiro, Rodolfo C. Pacagnella

**Affiliations:** I Universidade Estadual de Campinas Faculdade de Ciências Médicas Departamento de Tocoginecologia Campinas SP Brasil Universidade Estadual de Campinas . Faculdade de Ciências Médicas . Departamento de Tocoginecologia . Campinas , SP , Brasil; II Universidade de Pernambuco Centro Universitário Integrado de Saúde Amaury de Medeiros Recife PE Brasil Universidade de Pernambuco . Centro Universitário Integrado de Saúde Amaury de Medeiros . Recife , PE , Brasil

**Keywords:** Infant, Premature, Cervix Uteri, Mass Screening

## Abstract

**OBJECTIVE:**

To perform an economic cost analysis of the implementation of a short cervix screening program to reduce preterm birth in singleton pregnancies in a short-term time horizon.

**METHODS:**

We performed a cost-benefit economic analysis using the P5 trial database, a randomized multicenter clinical trial for prevention of preterm birth. Data collection was conducted from July 2015 to March 2019 in 17 different Brazilian hospitals. We conducted a cost analysis for universal cervical screening in singleton pregnancies between 18 weeks and 22 weeks plus 6 days. In subjects with a cervical length ≤ 25 mm, the analysis incorporated the costs of administering 200 mg/day of vaginal progesterone prophylactically until 36 weeks gestation. These findings were subsequently compared with the economic implications of forgoing cervical screening. The time horizon comprised from birth to 10 weeks postpartum. The outcome was measured monetarily in Brazilian real (R$) from the perspective of the Unified Health System.

**RESULTS:**

Among 7,844 women, 6.67% (523) had a cervix ≤ 25 mm. The cost of screening with transvaginal ultrasound and vaginal progesterone for prevention of births with < 34 weeks was estimated at R$ 383,711.36, while non-screening generated an estimated additional cost of R$ 446,501.69 (related to the 29 non-screened preterm deliveries). Thus, screening and prophylaxis would generate a final cost reduction of R$ 62,790.33, constituting a possible cost-benefit strategy.

**CONCLUSION:**

Universal short cervix screening for preterm birth has lower costs compared to non-screening within a short-term time horizon, which suggests an interesting benefit-cost ratio. Future studies should consider the cost-effectiveness of prophylactic treatment using sensitivity analyses in different scenarios within the Brazilian health system, as well as analyses that consider the long-term costs associated with preterm births, to robustly justify the implementation of a short cervix screening program.

## INTRODUCTION

Preterm birth affects one in ten births worldwide, and the pursuit of a reduction in this incidence remains one of the major challenges of medicine ^1^ . Spontaneous preterm birth (SPB) can be predicted by identifying a short cervix, preferably evidenced by transvaginal ultrasound (TVUS) in the second trimester of pregnancy ^2^ . Some authors advocate universal screening of pregnant women between 18 and 24 weeks in order to identify women at increased risk of preterm birth and implement therapies to prevent preterm delivery ^3 , 4^ .

Among the interventions to prevent preterm birth, progesterone is a widely used treatment to reduce preterm delivery, with few side effects and good adherence ^5^ . An individual patient data (IPD) meta-analysis of 2018 found that the use of vaginal progesterone reduced SPB with < 34 weeks by 28% (RR: 0.72, 95%CI 0.55–0.95) among women with short cervix (≤ 25mm) and estimated a number needed for treatment of 18 ^6^ . The most recent IPD meta-analysis on the use of progesterone (EPPPIC study) also found benefit with prophylactic use of vaginal progesterone and identified a reduction of preterm delivery with < 34 weeks by 22% ^7^ .

After the EPPPIC study, the Society of Maternal-Fetal Medicine and the American College of Obstetricians and Gynecologists reaffirmed the prophylactic use of progesterone in singleton pregnancy women with a cervix length ≤ 25 mm diagnosed between 18 0/7 and 22 6/7 weeks of gestation ^8^ . Several analyses show that universal screening has a good benefit-cost ratio when compared to not performing TVUS or offering it only when patients have a previous history of preterm delivery. However, there is still controversy as to whether or not to perform universal screening for short cervix ^11^ . Since these economic results vary depending on the prevalence of preterm birth in each population and on the transfers made by the health care provider, the results of the economic analyses of implementing or not a screening program depend on the country where the analysis is carried out; thus requiring decisions to be individualized.

In the world ranking of preterm birth, Brazil is in ninth position in absolute numbers and with 11.2% of preterm births, being directly responsible for 2.2% of all preterm births in the world ^1^ . According to the World Health Organization, there are 279,300 preterm births/year in Brazil ^14^ . In 2014, the Brazilian EMIP study involving 5,296 Brazilian pregnant women found an incidence of 12.3% premature births, with 2/3 of this total being SPBs ^15^ . The average cost of care required per preterm birth is estimated to be USD 1,427 for Brazil ^16^ . Given this high incidence of preterm births, it is necessary to project the costs of universal screening in the Brazilian population. Currently, Brazil does not have a screening program that is universal or geared toward patients with a history of preterm birth, and progesterone, still the best therapeutic option for the prevention of preterm birth, is not included in the group of standardized drugs provided by the Unified Health System (SUS).

In this sense, the objective of this study is to evaluate whether there is an economic benefit in the implementation of a cervical length screening program during pregnancy, followed by prophylactic treatment with vaginal progesterone of women then identified with a higher risk, compared to the costs associated with premature births, if not screened.

## METHOD

This is a cost-benefit ratio economic analysis, in which we compare the two scenarios of economic consequences: the implementation of a universal screening program for preterm birth *versus* no screening. By using a model where patients have 100% coverage and adherence to the proposed treatments, we estimate the costs of a universal screening program for preterm birth with the implementation of a well-established preventive treatment for all identified women at increased risk of preterm delivery (cervix ≤ 25mm) *versus* the cost linked to preterm deliveries that were not avoided due to the option of not screening. The outcome was measured monetarily using the Brazilian real (R$) as the reference currency and considering the values of the year 2021, from the perspective that the health care provider is SUS.

This study is a secondary analysis of the P5 Study on Pessary and Progesterone in Preterm Birth Prevention ^17^ . P5 is a randomized clinical trial with Brazilian pregnant women with cervical length ≤ 30 mm, identified through TVUS between 18–22 weeks of gestation. Coordinated by the Universidade Estadual de Campinas (UNICAMP) and involving 17 reference maternity hospitals in Brazil in three regions, P5 evaluated the effectiveness of isolated vaginal progesterone in relation to vaginal progesterone associated with cervical pessaries in the prevention of preterm delivery in pregnant women with short cervix. Data collection of the first phase of this study ( *screening* with TVUS) took place between July 2015 and March 2019. Cervix measurement was performed by an experienced professional sonographer following the protocol of the Fetal Medicine Foundation and with strict control of images and technical corrections when necessary, using a standardized device. All data were included in the P5 trial online database. In the second phase of the study (clinical trial), pregnant women with a cervix ≤ 30 mm were randomized into two groups: one group received vaginal progesterone 200 mg/day + cervical pessary and the other group received only vaginal progesterone 200 mg/day. The pregnant women in the clinical trial were followed up to 10 weeks after the probable date of delivery. The P5 study was evaluated and approved by the Unicamp Reasearch Ethics Commission (Opinion 1.082.011) and also by the *Comissão Nacional de Ética em Pesquisa* (National Research Ethics Commission – CONEP) (Opinion 1.055.555), and registered in the Brazilian registry of clinical trials (Trial registration RBR-3t8prz).

To estimate the number of women who would be identified and treated, we targeted pregnant women with a singleton pregnancy. Those with a uterine cervix ≤ 25 mm, identified through TVUS during the second trimester (18–22 weeks, the recommended interval for the test), are at an increased risk of preterm delivery before 34 weeks. Consequently, they should receive 200 mg/day of vaginal progesterone as a preventive treatment for preterm birth. Based on a recent meta-analysis on the subject, we consider as a reference that the number needed for treatment in this group is 18, that is, for every 18 pregnant women with a cervix ≤ 25 mm who receive progesterone among those identified with a cervix ≤ 25 mm up to 36 weeks of gestation, one SPB < 34 weeks is avoided ^18^ .

The P5 database contains the screening records of 7,892 women with singleton pregnancies, with 48 of whom lacked information regarding cervical measurement. Among the 7,844 women with complete screening data, the incidence of cervix ≤ 25 mm was 6.67%, a total of 523 patients. Following the literature information that for every 18 patients treated with vaginal progesterone we could reduce one SPB < 34 weeks, we can assume that universal screening followed by progesterone prophylaxis could reduce in our sample a total of 29 preterm deliveries < 34 weeks. To estimate the costs associated with treatment, we assumed in this model that treatment with vaginal progesterone 200 mg/day will be accepted and followed optimally by the patients identified from the 20th week to the 36th week of pregnancy.

Due to the limitation of data in the literature that could help us identify the average cost of a SPB < 34 weeks in Brazil, we used our P5 trial database to calculate how much a preterm birth would cost based on the average value found in our sample of preterms births with < 34 weeks among the women with a cervix ≤ 25 mm. In constructing this value, we considered the costs of inputs and procedures from birth to the first 10 weeks after the probable date of delivery, including possible neonatal readmissions, according to the follow-up period of the research subjects defined by the P5 trial protocol to identify persistent neonatal pathological conditions ( Table 1 ). We did not consider in the analysis the base and infrastructure costs for screening with TVUS in mid-pregnancy and full-term birth—admission of the newborn and care of the neonatologist in the delivery room. The values of the procedures are part of the table of the SUS Procedures, Medicines, and OPM Table Management System (SIGTAP). For medications, we used the *Agência Nacional de Vigilância Sanitária* (National Health Surveillance Agency – ANVISA) market regulation table—Drug Price Lists ^19^ . To estimate the value of daily treatment with vaginal progesterone, considering that it is not available in SUS among standardized drugs, we carried out a price survey including five large drugstore chains operating in Brazil and used the average value of one 200 mg progesterone capsule for vaginal use.

**Table 1:** Variables and units of value considering different sources.

Cost	Unit	Value R$	Source
Screening			
Transvaginal ultrasound	Procedure	24.2	SIGTAP
Prophylaxis			
Vaginal progesterone 200 mg/day	Day	3.31	Drug Stores
Birth			
Admission			
Infirmary	Day	137.2	SIGTAP
Semi-intensive	Day	180.0	SIGTAP
Intensive Care Unit	Day	508.63	SIGTAP
Drugs during hospitalization			
Pulmonary surfactant	Unit	519.74	ANVISA
MgSo4	Treatment	4.67	ANVISA
Antibiotics	Treatment	31.91	ANVISA
Phenobarbital	Treatment	0.76	ANVISA
Tests			
X-Ray	Procedure	6.88	SIGTAP
Ultrasound (transfontanelle)	Procedure	24.2	SIGTAP
Computed tomography	Procedure	97.44	SIGTAP
Neonatal readmission (< 10 weeks of delivery)	Unit	109.24	SIGTAP

SIGTAP: *Sistema de Gerenciamento da Tabela de Procedimentos, Medicamentos e OPM do SUS* (SUS Procedures, Medicines and OPM Table Management System); ANVISA: *Agência Nacional de Vigilância Sanitária* (National Health Surveillance Agency).

## RESULTS

In the calculation to estimate the average cost of SPB < 34 weeks, of the 41 pregnant women in the clinical trial with a cervix length of ≤ 25 mm, all experienced spontaneous preterm delivery (SPB) before 34 weeksSPB. Of these, 10 pregnancies had an adverse outcome with fetal or neonatal death. A total of five pregnancies evolved to fetal death and two newborns evolved to neonatal death with less than 24 hours of life; therefore, these seven births did not enter the cost assessment because there was no record of the costs associated with these early deaths. Thus, a total of 34 newborns were analyzed to calculate the average cost of preterm birth. Of these, 28 were considered with adverse outcome (82.35%), having presented some type of neonatal morbidity and three of them evolved to neonatal death during the follow-up period of the study. The average cost of SPB up to 10 weeks after the probable date of delivery was estimated at R$ 15,396.61 per newborn, which would give a total of R$ 446,501.69 associated with non-screening, that is, the cost of 29 SPBs < 34 weeks that were not avoided ( Table 2 ). In the P5 Study screening sample with data for 7,844 women, the mean uterine cervix was 36.9 mm and the incidence of cervix ≤ 25 mm was 6.67% (523 pregnant women). A total cost for universal screening of the entire sample was estimated at R$ 189,824.80, and a total cost for prophylactic treatment with progesterone of 523 pregnant women with short cervix was estimated at R$ 193,886.56; totaling R$ 383,711.36 to avoid 29 preterm births. This result identified that universal screening associated with prophylactic treatment with vaginal progesterone presented a good benefit-cost ratio in the short term, with a total cost reduction estimated at R$ 62,790.33 (screening + prophylaxis x SPB < 34 – scenario a – lower variation in the price of progesterone, absolute difference: -R$ 69,233.69; scenario b – higher variation in the price of progesterone: -R$ 51,660.89) ( Table 3 ).

**Table 2:** Estimated average cost of one SPB < 34 weeks among women with a cervix ≤ 25 mm participating in the P5 trial.

SPB < 34 cost	Unit	Value R$	Total units	Average cost per newborn
Admission				
Infirmary	Day	137.2	191	770.74
Semi-intensive	Day	180.0	75	397.06
Intensive Care Unit	Day	508.63	928	13,882.61
Drugs during hospitalization				
Pulmonary surfactant	Unit	519.74	16	244.58
MgSo4	Treatment	4.67	14	1.92
Antibiotics (diagnosed sepsis)	Treatment	31.91	3	2.82
Phenobarbital	Treatment	0.76	4	0.09
Tests				
X-Ray	Procedure	6.88	13	2.63
Ultrasound (transfontanelle)	Procedure	24.2	19	13.52
Computed tomography	Procedure	97.44	3	8.6
Neonatal readmission (< 10 weeks)	Unit	109.24 + total daily fees	1	72.04
Cost of SPB < 34 birth				15,396.61

Note: estimated costs in Brazilian Real (BRL).

SPB: spontaneous preterm delivery.

Total N = 34 spontaneous preterm deliveries < 34 weeks occurred in the P5 trial with possible valuation.

**Table 3:** Costs of universal screening + SPB < 34 prophylaxis x costs related to non-screening.

Variables	Cost of unit	Cost per pregnant woman	Total cost
Ultrasound (n = 7,844)	R$ 24.20	R$ 24.20	R$ 189,824.80
Progesterone (n = 523)	R$ 3.31	R$ 370.72	R$ 193,886.56
Total (TVUS + progesterone)			R$ 383,711.36
		Cost per SPB < 34	
Cost per SPB < 34 (n = 29)		15,396.61	R$ 446,501.69
Absolute difference (screening + prophylaxis x no screening)			-R$ 62,790.33

SPB: spontaneous preterm delivery; TVUS: transvaginal ultrasound.

Among the analyzed costs related to preterm birth, the main responsible for the final cost was the newborn’s length of stay in the ICU, with an average cost of R$ 13,882.61 per birth. Of the 34 newborns analyzed, 47% required pulmonary surfactant. Additionally, 41.2% received magnesium sulfate for neuroprotection. Meanwhile, 55.9% underwent a transfontanelle ultrasound. Lastly, 11.2% experienced seizure episodes that necessitated the use of an anticonvulsant.

## DISCUSSION

The analysis estimated that implementing a screening program with TVUS in the second trimester of pregnancy to identify pregnant women with a cervix measure ≤ 25 mm, followed by prophylactic treatment with vaginal progesterone up to 36 weeks of gestation, costs less than the average of a SPB below 34 weeks. Considering the effectiveness of progesterone previously reported in the literature, its ideal use and the existence of an existing infrastructure for screening, the universal screening strategy for the prevention of preterm birth represents a saving of R$ 2,165.18 for each preterm birth avoided in a short-term projection, in which only the first 10 weeks after the probable date of delivery were considered.

Previous studies have advocated universal screening considering the positive economic repercussions ^20 , 21^ . An American article considering a prevalence of 8% of cervixes ≤ 25 mm and prophylaxis with vaginal progesterone estimated that universal screening would have prevented 30,545 preterm deliveries < 34 weeks in 2013 ^22^ . However, it is important to note that preterm birth of < 34 weeks is associated with long-term cost-generating consequences, such as cognitive, respiratory, cardiovascular, auditory and visual sequelae, which were not measured in this study ^23^ , but may further influence on the economic impact and cost reduction to the health care provider if considered ^24 , 25^ . Moreover, the reduction of preterm birth-related fetal and neonatal mortality, gain in quality of life for children and families, and reduced loss of productivity for parents or guardians were also not part of this study ^26^ . International studies that included these factors in their analysis indicated that universal screening is cost-effective in preventing preterm birth below 34 weeks ^27 , 28^ .

Our study used the prevalence of cervix ≤ 25 mm of a large sample of Brazilian pregnant women from different regions of the country. Preterm births used as a reference for the average cost of preterm birth were followed during the valuation period, providing complete data regarding the costs involved in the analyzed time horizon. However, as a limitation, this study did not consider in the analysis the costs prior to performing the ultrasound examination, such as the costs related to the ultrasound device, clinic, and physical infrastructure.

In this regard, we consider that it would be possible to use the same device reserved for routine obstetric ultrasound and the same probe used during the obstetric ultrasound of the first trimester, not significantly burdening the costs related to equipment and infrastructure in centers that already perform routine prenatal ultrasound exams. The examination can and should be performed at the same time when the pregnant woman seeks the health care unit for obstetric or morphological GUS in the second trimester and by the same professional, considering an increase of 5–10 minutes in the examination time. Information from the National Health Survey (PNS) carried out in Brazil in 2013 with women from all over the national territory showed that 99.7% of the women interviewed had undergone some ultrasound during pregnancy, which makes it clear that this prenatal examination is already incorporated into the health care culture of the Brazilian population and that it has easy access ^29^ .

However, implementation of the screening program would require trained professionals to perform cervical measurement. This control in the quality of the exam provided is feasible, since the technique for measuring the cervix is simple, with a learning curve that requires little time. The training for this technique is available for free and online on the Fetal Medicine Foundation platform, with evaluation and audit of images at the end of the course; therefore, it is accessible to professionals without significant cost increase ^30^ .

Notably, in order to estimate the cost of preterm delivery, we considered the costs related to the first 10 weeks after the probable date of delivery; additionally, the costs related to the five fetal deaths and two neonatal deaths of our sample were not added. We consider this a limitation of our study. A long-term analysis could provide even more impactful results as to the cost-benefit ratio of implementing the screening program.

Our study also presents the limitation of not considering the costs associated with the follow-up of women identified as at risk of preterm birth. It is true that, after a short cervix is identified, each patient will form their perception of risk and this can lead to more anxiety, stress, prenatal consultations, and work-related losses. At the same time, building a good doctor-patient relationship—which should be the premise of all medical care and which does not add to the final cost—can provide the necessary clarifications and guidance to reduce this state of vulnerability and facilitate adherence to the proposed treatment ^31^ .

Thus, we can consider this analysis as a preliminary cost study, a first step towards a more attentive look into the possibility of implementing TVUS for uterine cervix measurement in the second trimester as a screening program for preterm birth in Brazil. We understand that the decision to use the costs of preterm birth treatment according to the SUS table (underestimated in relation to the actual cost) and the values of short cervix treatment according to CMED/ANVISA (market prices, higher than the prices of a possible direct acquisition), ultimately overestimate the costs of prevention and underestimate the costs of treatment and, therefore, underestimate the reduction and costs with screening. Even with this consideration, we found a cost reduction favorable to screening.

However, the development of more robust results will require cost-effectiveness analysis with the modeling of several other factors that directly influence the incremental cost result, such as the acceptability to undergoing the screening, the real adherence to the treatment offered, and the work-related losses of women at higher risk of preterm delivery, enabling the estimation of values more consistent with reality through sensitivity analyses.

## CONCLUSION

Considering the above, we conclude that universal screening of short cervix for prevention of preterm birth with TVUS in the second trimester of pregnancy, associated with prophylaxis with vaginal progesterone for pregnant women identified with a cervix ≤ 25 mm, presents lower costs in relation to non-screening within a short-term time horizon, which suggests an interesting benefit-cost ratio in the implementation of universal screening of short cervix for prevention of preterm birth in Brazil. New studies that also consider the cost-effectiveness of prophylactic treatment using sensitivity analyses in different scenarios within the Brazilian health care system are necessary to robustly justify the screening of preterm birth.

## References

[1] Chawanpaiboon S, Vogel JP, Moller AB, Lumbiganon P, Petzold M, Hogan D (2019). Global, regional, and national estimates of levels of preterm birth in 2014: a systematic review and modelling analysis. Lancet Glob Health.

[2] Iams JD, Goldenberg RL, Meis PJ, Mercer BM, Moawad A, Das A, National Institute of Child Health and Human Development Maternal Fetal Medicine Unit Network (1996). The length of the cervix and the risk of spontaneous premature delivery. N Engl J Med.

[3] Liu CZ, Ho N, Nguyen AD, Lehner C, Sekar R, Amoako AA (2021). The risk of preterm delivery and pregnancy outcomes in women with asymptomatic short cervix: a retrospective cohort study. J Matern Neonatal Med.

[4] Souka AP, Papastefanou I, Pilalis A, Kassanos D, Papadopoulos G (2019). Implementation of universal screening for preterm delivery by mid-trimester cervical-length measurement. Ultrasound Obstet Gynecol.

[5] Figo Working Group On Best Practice In Maternal-Fetal Medicine, International Federation of Gynecology and Obstetrics (2015). Best practice in maternal-fetal medicine. Int J Gynaecol Obstet Off organ Int Fed Gynaecol Obstet.

[6] Romero R, Conde-Agudelo A, Fonseca E, O’Brien JM, Cetingoz E, Creasy GW (2018). Vaginal progesterone for preventing preterm birth and adverse perinatal outcomes in singleton gestations with a short cervix: a meta-analysis of individual patient data. Am J Obstet Gynecol.

[7] Stewart LA, Simmonds M, Duley L, Llewellyn A, Sharif S, Walker RA (2021). Evaluating Progestogens for Preventing Preterm birth International Collaborative (EPPPIC): meta-analysis of individual participant data from randomised controlled trials. Lancet.

[8] Society for Maternal-Fetal Medicine Publications Committee, Vincenzo Berghella (2012). Progesterone and preterm birth prevention: translating clinical trials data into clinical practice. Am J Obstet Gynecol.

[9] SMFM Statement (2020). use of 17-alpha hydroxyprogesterone caproate for prevention of recurrent preterm birth. Am J Obstet Gynecol.

[10] Prediction and prevention of spontaneous preterm birth (2021). Prediction and ACOG Practice Bulletin, Number 234. Obstet Gynecol.

[11] Rozenberg P (2016). Is universal screening for cervical length among singleton pregnancies with no history of preterm birth justified?. J Gynecol Obstet Biol Reprod (Paris).

[12] Peixoto AB, Caldas TMC, Tahan LA, Petrini CG, Martins WP, Costa FD (2017). Second trimester cervical length measurement for prediction spontaneous preterm birth in an unselected risk population. Obstet Gynecol Sci.

[13] Campbell S (2011). Universal cervical-length screening and vaginal progesterone prevents early preterm births, reduces neonatal morbidity and is cost saving: doing nothing is no longer an option. Ultrasound Obstet Gynecol.

[14] Dean S V, Mason E, Howson CP, Lassi ZS, Imam AM, Bhutta ZA (2013). Born too soon: care before and between pregnancy to prevent preterm births: from evidence to action. Reprod Health.

[15] Passini R, Cecatti JG, Lajos GJ, Tedesco RP, Nomura ML, Dias TZ (2014). Brazilian multicentre study on preterm birth (EMIP): prevalence and factors associated with spontaneous preterm birth. PLoS One.

[16] Desgualdo CM, Riera R, Zucchi P (2011). Cost estimate of hospital stays for premature newborns in a public tertiary hospital in Brazil. Clinics (São Paulo).

[17] Pacagnella RC, Silva T, Cecatti JG, Passini-Jr R, Fanton T, Borovac-Pinheiro A (2021). 2 Pessary plus progesterone to prevent preterm birth in women with a short cervix (P5 trial). Am J Obstet Gynecol.

[18] Conde-Agudelo A, Romero R, Fonseca E, O’Brien JM, Cetingoz E, Creasy GW (2018). Vaginal progesterone is as effective as cervical cerclage to prevent preterm birth in women with a singleton gestation, previous spontaneous preterm birth, and a short cervix: updated indirect comparison meta-analysis. Am J Obstet Gynecol.

[19] Agência Nacional de Vigilância Sanitária (2021). Listas de preços de medicamentos.

[20] Cahill AG, Odibo AO, Caughey AB, Stamilio DM, Hassan SS, Macones GA (2010). Universal cervical length screening and treatment with vaginal progesterone to prevent preterm birth: a decision and economic analysis. Am J Obstet Gynecol.

[21] Werner EF, Hamel MS, Orzechowski K, Berghella V, Thung SF (2015). Cost-effectiveness of transvaginal ultrasound cervical length screening in singletons without a prior preterm birth: an update. Am J Obstet Gynecol.

[22] Conde-Agudelo A, Romero R (2016). Vaginal progesterone to prevent preterm birth in pregnant women with a sonographic short cervix: clinical and public health implications. Am J Obstet Gynecol.

[23] Luu TM, Rehman Mian MO, Nuyt AM (2017). Long-term impact of preterm birth: neurodevelopmental and physical health outcomes. Clin Perinatol.

[24] Jacob J, Lehne M, Mischker A, Klinger N, Zickermann C, Walker J (2017). Cost effects of preterm birth: a comparison of health care costs associated with early preterm, late preterm, and full-term birth in the first 3 years after birth. Eur J Health Econ.

[25] Kim HJ, Jo MW, Bae SH, Yoon SJ, Lee JY (2019). Measuring the burden of disease due to preterm birth complications in Korea Using Disability-Adjusted Life Years (DALY). Int J Environ Res Public Health.

[26] Kyu HH, Abate D, Abate KH, Abay SM, Abbafati C, Abbasi N (2018). Global, regional, and national disability-adjusted life-years (DALYs) for 359 diseases and injuries and healthy life expectancy (HALE) for 195 countries and territories, 1990-2017: a systematic analysis for the Global Burden of Disease Study 2017. Lancet.

[27] McCurdy RJ, Baxter JK (2020). Universal cervical length screening with a cervicometer to prevent preterm birth <34 weeks: a decision and economic analysis. J Matern neonatal Med.

[28] Crosby DA, Miletin J, Semberova J, Daly S (2016). Is routine transvaginal cervical length measurement cost-effective in a population where the risk of spontaneous preterm birth is low?. Acta Obstet Gynecol Scand.

[29] Mario DN, Rigo L, Boclin KL, Malvestio LM, Anziliero D, Horta BL (2019). Quality of Prenatal Care in Brazil: National Health Research 2013. Cien Saude Colet.

[30] The Fetal Medicine Foundation (2021). The FMF certification cervical assessment.

[31] Silva TV, Bento SF, Katz L, Pacagnella RC (2021). “Preterm birth risk, me?” Women risk perception about premature delivery: a qualitative analysis. BMC Pregnancy Childbirth.

