# Supplementary material for: Estimates of avoided costs attributed to ashort cervix screening program to preventpreterm birth from the perspective of theUnified Health System (SUS)
Source: Rev Saude Publica. 2023 Oct 30;57:87. doi: 10.11606/s1518-8787.2023057004376 (PMC10653101; doi:10.11606/s1518-8787.2023057004376)
Supplement: Translation

# Translation: Estimativa de custos evitados atribuídos a um programa de rastreio de colo curto para prevenção da prematuridade na perspectiva do SUS

**Authors:** Thais V. Silva, Anderson Borovac-Pinheiro, Rodolfo C. Pacagnella

**Affiliations:** I Brasil Universidade Estadual de Campinas. Faculdade de Ciências Médicas. Departamento de Tocoginecologia. Campinas, SP, Brasil; II Brasil Universidade de Pernambuco. Centro Universitário Integrado de Saúde Amaury de Medeiros. Recife, PE, Brasil

**Keywords:** Recém-Nascido Prematuro, Colo do Útero, Programas de Rastreamento

## Abstract

**OBJETIVO:**

Realizar uma análise econômica de custo da implementação de um programa de rastreio de colo curto para redução da prematuridade em gestações únicas num horizonte temporal de curto prazo.

**MÉTODOS:**

Realizamos uma análise econômica do tipo custo-benefício utilizando o banco de dados do P5 trial, um ensaio clínico multicêntrico randomizado para prevenção da prematuridade. A coleta de dados ocorreu de julho de 2015 a março de 2019 em 17 diferentes hospitais do Brasil. Comparamos os custos do rastreamento universal em mulheres com gestação única de 18 a 22 semanas e 6 dias associado à progesterona vaginal profilática 200 mg/dia até 36 semanas naquelas com colo ≤ 25 mm com os do não rastreamento. O horizonte temporal foi do nascimento até 10 semanas após o parto. O desfecho foi medido monetariamente em real brasileiro (R$) na perspectiva do Sistema Único de Saúde.

**RESULTADOS:**

Entre 7.844 mulheres, 6,67% (523) apresentaram colo ≤ 25mm. O custo do rastreio com a ultrassonografia transvaginal mais a progesterona vaginal para prevenção de nascimentos < 34 semanas foi estimado em R$ 383.711,36, enquanto o não rastreamento gerou custo adicional estimado de R$ 446.501,69 (relacionado aos 29 partos prematuros não rastreados). Assim, o rastreamento mais a profilaxia geraria uma redução de custo final de R$ 62.790,33, apresentando-se como uma possível estratégia de custo-benefício.

**CONCLUSÃO:**

O rastreamento universal de colo curto para prematuridade apresenta menores custos em relação ao não rastreamento dentro de um horizonte temporal de curto prazo, o que sugere uma interessante relação de custo *versus* benefício. Novos estudos que considerem a custo-efetividade do tratamento profilático utilizando-se de análises de sensibilidade em diferentes cenários dentro do sistema de saúde brasileiro, assim como análises que considerem os custos de longo prazo atrelados ao nascimento prematuro, são necessários para justificar com robustez a implementação de um programa de rastreamento.

## INTRODUÇÃO

O parto pré-termo afeta um a cada dez nascimentos em todo mundo, e a busca pela redução dessa incidência continua sendo um dos grandes desafios da medicina ^1^ . A predição do parto pré-termo espontâneo (PPE) pode ser feita pela identificação do colo uterino curto, evidenciado preferencialmente através da ultrassonografia transvaginal (USTV) no segundo trimestre de gestação ^2^ . Alguns autores defendem a realização de um rastreio universal das gestantes entre 18 e 24 semanas a fim de identificar as mulheres sob risco aumentado de prematuridade e implementar terapias para prevenir o parto pré-termo ^3 , 4^ .

Entre as intervenções para prevenir a prematuridade, a progesterona é um tratamento bastante utilizado para redução do parto pré-termo, com poucos efeitos colaterais e boa adesão ^5^ . Uma *individual patient data* (IPD) metanálise de 2018 identificou que o uso da progesterona vaginal reduziu o PPE < 34 semanas em 28% (RR: 0.72, IC95% 0.55–0.95) entre mulheres com colo uterino curto (≤ 25 mm) e estimou um número necessário para tratamento de 18 ^6^ . A mais recente IPD metanálise sobre o uso da progesterona (EPPPIC *study* ) também encontrou benefício com o uso profilático da progesterona vaginal e identificou uma redução de parto pré-termo < 34 semanas em 22% ^7^ .

Após o EPPPIC *study* , a Sociedade de Medicina Materno-Fetal e o American College of Obstetricians and Gynecologists reafirmaram o uso profilático da progesterona em mulheres com gestação única e colo uterino com comprimento ≤ 25mm diagnosticado entre 18 0/7 e 22 6/7 semanas de gestação ^8^ . Muitas análises mostram que o rastreio universal tem uma boa relação custo-benefício quando comparado à conduta de não realizar a USTV ou oferecer apenas quando as pacientes apresentam história prévia de parto pré-termo. Entretanto, ainda existe controvérsia quanto a realizar ou não o rastreio universal para colo curto ^11^ . Como esses resultados econômicos variam a depender da prevalência do parto pré-termo em cada população e dos repasses feitos pelo provedor de saúde, os resultados das análises econômicas de implementar ou não um programa de rastreio variam a depender do país onde a análise é realizada e, portanto, as decisões precisam ser individualizadas.

No ranking mundial de prematuridade, o Brasil encontra-se na nona posição em números absolutos e com 11,2% de nascimentos prematuros, sendo responsável direto por 2,2% de todos os nascimentos prematuros no mundo ^1^ . Segundo a Organização Mundial da Saúde, nascem 279.300 prematuros/ano em nosso país ^14^ . Em 2014, o estudo brasileiro EMIP envolvendo 5.296 gestantes brasileiras encontrou uma incidência de 12,3% de partos prematuros, com 2/3 desse total sendo PPE ^15^ . Estima-se que o custo médio dos cuidados necessários por nascimento prematuro seja de $ 1.427 dólares para o Brasil ^16^ . Diante dessa alta incidência de partos prematuros, é necessário projetar os custos do rastreamento universal na população brasileira. Atualmente o Brasil não possui um programa de rastreio universal nem direcionado para pacientes com histórico de prematuridade, e a progesterona, ainda hoje a melhor opção terapêutica para prevenção de prematuridade, não faz parte do grupo de medicamentos padronizados oferecidos pelo Sistema Único de Saúde (SUS).

Nesse sentido, o objetivo deste estudo é avaliar se existe benefício econômico na implementação de um programa de rastreamento do comprimento cervical durante a gestação, seguido pelo tratamento profilático com progesterona vaginal das mulheres então identificadas com maior risco, em comparação aos custos atrelados aos nascimentos prematuros, se optado pelo não rastreamento.

## MÉTODO

Esta é uma análise econômica do tipo custo-benefício na qual comparamos os dois cenários de consequências econômicas: a implementação de um programa de rastreio universal para prematuridade *versus* o não rastreamento. Utilizando-nos de um modelo em que as pacientes apresentam 100% de cobertura e adesão aos tratamentos propostos, valoramos os custos de um programa de rastreio universal para prematuridade com implementação de um tratamento preventivo bem estabelecido para todas as mulheres identificadas com risco aumentado de parto pré-termo (colo ≤ 25 mm) *versus* o custo atrelado aos partos prematuros não evitados pela opção do não rastreamento. O desfecho foi medido monetariamente usando como moeda de referência o real brasileiro (R$) e considerando os valores do ano 2021, na perspectiva de que o provedor é o SUS.

Este estudo é uma análise secundária do Estudo P5 – Pessário e Progesterona na Prevenção do Parto Pré-termo ^17^ . O P5 é um ensaio clínico randomizado com gestantes brasileiras com o comprimento cervical ≤ 30 mm, identificadas a partir de USTV entre 18–22 semanas de gestação. Coordenado pela Universidade Estadual de Campinas (Unicamp) e envolvendo 17 maternidades de referência do Brasil em três regiões, o P5 avaliou a efetividade da progesterona vaginal isolada em relação à progesterona vaginal associada ao pessário cervical na prevenção do parto pré-termo em gestantes com colo curto. A coleta de dados da primeira fase desse estudo ( *screening* com USTV) ocorreu entre julho de 2015 e março de 2019. A medida do colo foi realizada por profissional ultrassonografista experiente seguindo o protocolo da Fetal Medicine Foundation e com rigoroso controle de imagens e correções técnicas quando necessário, utilizando um aparelho padronizado. Todos os dados foram incluídos no banco de dados on-line do P5 trial. Na segunda fase do estudo (ensaio clínico) gestantes com colo ≤ 30 mm foram randomizadas em dois grupos: um grupo recebeu progesterona 200 mg/dia por via vaginal + pessário cervical e o outro grupo recebeu apenas a progesterona vaginal 200 mg/dia. As gestantes do ensaio clínico foram seguidas até 10 semanas após a data provável do parto. O estudo P5 foi avaliado e aprovado pelo CEP da Unicamp (Parecer 1.082.011) e também pela Comissão Nacional de Ética em Pesquisa (Conep) (Parecer 1.055.555), e cadastrado no registro brasileiro de ensaios clínicos (Trial registration RBR-3t8prz).

Para estimar o número de mulheres a serem identificadas e tratadas, partimos do pressuposto de que gestantes com gravidez única (população-alvo), com colo uterino ≤ 25 mm identificado através de USTV no segundo trimestre de gestação (18–22 sem – intervalo de recomendação da realização do exame) têm risco aumentado de parto pré-termo < 34 semanas e, portanto, devem receber progesterona vaginal 200mg/dia como tratamento preventivo para prematuridade. A partir de recente metanálise sobre o tema, consideramos como referência que o número necessário para tratamento neste grupo é 18, ou seja, a cada 18 gestantes com colo ≤ 25mm que recebem progesterona entre as identificadas com colo uterino ≤ 25mm até 36 semanas de gestação, um PPE < 34 semanas é evitado ^18^ .

O banco de dados do P5 contém o registro de rastreamento de 7.892 mulheres com gestação única, com 48 pacientes apresentando perda de informação com relação à medida do colo. Entre as 7.844 mulheres com dados completos do rastreamento, a incidência de colo ≤ 25mm foi de 6,67%, um total de 523 pacientes. Seguindo a informação da literatura de que a cada 18 pacientes tratadas com progesterona vaginal poderíamos reduzir um PPE < 34 semanas, podemos supor que o rastreamento universal seguido de profilaxia com progesterona poderia reduzir em nossa amostra um total de 29 partos prematuros < 34 semanas. Para estimar os custos associados ao tratamento, assumimos nesse modelo que o tratamento com progesterona vaginal 200 mg/dia será aceito e seguido de forma ideal pelas pacientes identificadas da 20ª semana até a 36ª semana de gestação.

Devido à limitação de dados na literatura que pudessem nos ajudar a identificar o custo médio de um PPE < 34 semanas nascido no Brasil, utilizamos nosso banco de dados do P5 trial para calcular quanto custaria um nascimento prematuro a partir do valor médio identificado em nossa amostra de prematuros < 34 semanas nascidos entre as mulheres com colo ≤ 25mm. Na construção desse valor, foram considerados os custos de insumos e procedimentos desde o nascimento até as 10 primeiras semanas após a data provável do parto, incluindo possíveis readmissões neonatais, conforme o período de seguimento dos sujeitos de pesquisa definido pelo protocolo do P5 trial de maneira a identificar condições patológicas neonatais persistentes ( Tabela 1 ). Não consideramos na análise os custos-base e de infraestrutura para rastreio com USTV na metade da gestação e nascimento a termo – admissão do recém-nascido e atendimento do neonatologista em sala de parto. Os valores dos procedimentos fazem parte da tabela do Sistema de Gerenciamento da Tabela de Procedimentos, Medicamentos e OPM do SUS (SIGTAP). Para medicações, utilizamos a tabela da Agência Nacional de Vigilância Sanitária (Anvisa) de regulação de mercado – Listas de Preços de Medicamentos ^19^ . Para estimar o valor do tratamento diário com a progesterona vaginal, tendo em vista que ela não é disponibilizada pelo SUS entre os medicamentos padronizados, realizamos um levantamento de preço incluindo cinco grandes redes de farmácias atuantes no Brasil e utilizamos o valor médio de uma cápsula de 200mg de progesterona para uso vaginal.

**Tabela 1:** Variáveis e unidades de valor considerando diferentes fontes.

Custo	Unidade	Valor R$	Fonte
Rastreio			
Ultrassonografia transvaginal	Procedimento	24,2	SIGTAP
Profilaxia			
Progesterona vaginal 200mg/dia	Dia	3,31	Farmácias
Nascimento			
Admissão			
Enfermaria	Dia	137,2	SIGTAP
Semi-intensiva	Dia	180,0	SIGTAP
Unidade de terapia intensiva	Dia	508,63	SIGTAP
Drogas durante internamento			
Surfactante pulmonar	Unidade	519,74	Anvisa
MgSo4	Tratamento	4,67	Anvisa
Antibióticos	Tratamento	31,91	Anvisa
Fenobarbital	Tratamento	0,76	Anvisa
Exames			
Raio X	Procedimento	6,88	SIGTAP
Ultrassonografia (transfontanela)	Procedimento	24,2	SIGTAP
Tomografia computadorizada	Procedimento	97,44	SIGTAP
Readmissão neonatal (< 10 semanas do parto)	Unidade	109,24	SIGTAP

SIGTAP: Sistema de Gerenciamento da Tabela de Procedimentos, Medicamentos e OPM do SUS; Anvisa: Agência Nacional de Vigilância Sanitária.

## RESULTADOS

No cálculo para estimar o custo médio do PPE < 34, 41 gestantes participantes do ensaio clínico com colo ≤ 25mm tiveram um PPE < 34 semanas. Destas, 10 gestações tiveram desfecho adverso com óbito fetal ou neonatal. Um total de cinco gestações evoluíram com óbito fetal e dois recém-nascidos evoluíram para óbito neonatal com menos de 24h de vida, portanto esses sete nascimentos não entraram na avaliação de custo por não haver registro dos custos associados a esses óbitos precoces. Dessa forma, um total de 34 recém-nascidos foram analisados para o cálculo do custo médio da prematuridade. Destes, 28 foram considerados como não saudáveis (82,35%), tendo apresentado algum tipo de morbidade neonatal e três deles evoluíram para óbito neonatal durante o período de seguimento do estudo. O custo médio do PPE até 10 semanas após a data provável do parto foi estimado em R$ 15.396,61 por recém-nascido, o que daria um total de R$ 446.501,69 associados ao não rastreamento, ou seja, o custo de 29 PPE < 34 semanas que não foram evitados ( Tabela 2 ).

**Tabela 2:** Custo médio estimado de um PPE < 34 semanas entre mulheres com colo ≤ 25 mm participantes do P5 trial.

Custo PPE < 34	Unidade	Valor R$	Total de unidades	Custo médio por recém-nascido
Admissão				
Enfermaria	Dia	137,2	191	770,74
Semi-intensiva	Dia	180,0	75	397,06
Unidade de terapia intensiva	Dia	508,63	928	13.882,61
Drogas durante internamento				
Surfactante pulmonar	Unidade	519,74	16	244,58
MgSo4	Tratamento	4,67	14	1,92
Antibióticos (sepse disgnosticada)	Tratamento	31,91	3	2,82
Fenobarbital	Tratamento	0,76	4	0,09
Exames				
Raio X	Procedimento	6,88	13	2,63
Ultrassonografia (transfontanela)	Procedimento	24,2	19	13,52
Tomografia computadorizada	Procedimento	97,44	3	8,6
Readmissão neonatal (< 10 sem.)	Unidade	109,24 + total de diárias	1	72,04
Custo do nascimento PPE < 34				15.396,61

Nota: custos estimados em real brasileiro (BRL).

PPE: parto pré-termo espontâneo.

N total = 34 nascimentos prematuros espontâneos < 34 semanas ocorridos no P5 trial possíveis de valoração.

Na amostra de rastreio do Estudo P5 com dados de 7.844 mulheres, a média de colo uterino foi 36,9mm e a incidência de colo ≤ 25mm foi 6,67% (523 gestantes). Estimamos um custo total para o rastreio universal de toda a amostra de R$ 189.824,80 e um custo total de tratamento profilático com progesterona de 523 gestantes com colo curto de R$ 193.886,56; perfazendo um total de R$ 383.711,36 para evitar 29 partos prematuros. Esse resultado identificou que o rastreio universal associado ao tratamento profilático com progesterona vaginal apresentou boa relação custo-benefício em curto prazo, com redução de custos totais estimada em R$ 62.790,33 (rastreio + profilaxia x PPE < 34 – cenário a – variação inferior do preço da progesterona: diferença absoluta: -R$ 69.233,69; cenário b – variação superior do preço da progesterona: -R$ 51.660,89) ( Tabela 3 ).

**Tabela 3:** Custos do rastreio universal + profilaxia para PPE < 34 x custos relacionados ao não rastreamento.

Variáveis	Custo da unidade	Custo por gestante	Custo total
Ultrassonografia (n = 7.844)	R$ 24,20	R$ 24,20	R$ 189.824,80
Progesterona (n = 523)	R$ 3,31	R$ 370,72	R$ 193.886,56
Total (UST + progesterona)			R$ 383.711,36
		Custo por PPE < 34	
Custo PPE < 34 (n = 29)		15.396,61	R$ 446.501,69
Diferença absoluta (rastreio + profilaxia x não rastreio)			-R$ 62.790,33

PPE: parto pré-termo espontâneo; UST: ultrassonografia transvaginal.

Entre os custos analisados relacionados ao nascimento prematuro, o maior responsável pelo custo final foi o tempo de internação do recém-nascido em UTI, com um custo médio de R$ 13.882,61 por nascimento. Entre os 34 recém-nascidos analisados, 47% necessitaram de surfactante pulmonar, 41,2% receberam sulfato de magnésio para neuroproteção, 55,9% realizaram ultrassonografia transfontanela e 11,2% apresentaram episódios convulsivos sendo necessário o uso de anticonvulsivante.

## DISCUSSÃO

A análise estimou que implementar um programa de rastreio com USTV no segundo trimestre de gravidez para identificação de gestantes com medida de colo ≤ 25 mm, seguido de tratamento profilático com progesterona vaginal até as 36 semanas de gestação, tem um custo inferior ao custo médio de um PPE abaixo de 34 semanas. Considerando a efetividade da progesterona relatada previamente em literatura, seu uso ideal e a existência de uma infraestrutura já existente para a realização do rastreamento, a estratégia de rastreio universal para prevenção da prematuridade representa uma economia de R$ 2.165,18 para cada parto pré-termo evitado numa projeção de curto prazo, na qual foram consideradas apenas as 10 primeiras semanas após a data provável do parto.

Estudos anteriores têm defendido o rastreio universal considerando as repercussões econômicas positivas ^20 , 21^ . Um artigo americano considerando uma prevalência de 8% de colos ≤ 25mm e a profilaxia com progesterona vaginal estimou que o rastreio universal teria prevenido 30.545 partos pré-termo < 34 semanas no ano de 2013 ^22^ . Entretanto, é importante lembrar que a prematuridade < 34 semanas está associada a consequências de longo prazo geradoras de custos, como sequelas cognitivas, respiratórias, cardiovasculares, auditivas e visuais, que não foram mensurados nesse estudo ^23^ , mas que poderiam trazer ainda mais impacto econômico e redução de gastos ao provedor de saúde se consideradas ^24 , 25^ . Mais ainda, também não fizeram parte deste estudo a redução da mortalidade fetal e neonatal associada à prematuridade, o ganho de qualidade de vida da criança e sua família, assim como a redução da perda de produtividade dos pais ou responsáveis pela criança ^26^ . Estudos internacionais que incluíram esses fatores em sua análise indicaram que o *screening* universal tem custo-benefício efetivo para prevenir prematuridade abaixo de 34 semanas ^27 , 28^ .

Nosso estudo utilizou a prevalência de colo ≤ 25mm de uma grande amostra de gestantes brasileiras de distintas regiões do país. Os nascimentos prematuros utilizados como referência para o custo-médio da prematuridade foram seguidos durante o período de valoração, permitindo dados completos quanto aos custos envolvidos no horizonte temporal analisado. Entretanto, como limitação, o presente estudo não considerou na análise os custos prévios à realização do exame de ultrassonografia, como os custos relacionados ao aparelho de ultrassonografia, clínica e infraestrutura física.

Sobre isso, consideramos que seria possível utilizar o mesmo aparelho reservado para ultrassonografia obstétrica de rotina e a mesma sonda utilizada durante a ultrassonografia obstétrica do primeiro trimestre, não onerando expressivamente os custos relacionados a aparelhagem e infraestrutura nos centros que já realizam exames ultrassonográficos de rotina de pré-natal. O exame pode e deve ser feito no mesmo momento em que a gestante procura a unidade de saúde para realização de USG obstétrica ou morfológica do segundo trimestre e pelo mesmo profissional, sendo considerado um acréscimo de 5–10 minutos no tempo de exame. Informação proveniente da Pesquisa Nacional em Saúde (PNS) realizada no Brasil em 2013 com mulheres de todo o território nacional mostrou que 99,7% das mulheres entrevistadas tinham realizado alguma ultrassonografia durante a gestação, o que deixa claro que esse exame do pré-natal já está incorporado na cultura de saúde da população brasileira e que apresenta facilidade de acesso ^29^ .

A implementação do programa de rastreio, todavia, necessitaria de profissionais treinados para realização da medida do colo uterino. Esse controle na qualidade do exame oferecido é factível, visto que a técnica para medida do colo é simples e com curva de aprendizado que demanda pouco tempo, sendo possível realizar treinamento on-line e gratuito na plataforma da Fetal Medicine Foundation sem custo, com avaliação e auditoria de imagens ao término do treinamento; portanto, disponível e acessível para capacitação dos profissionais sem expressivo acréscimo de custo ^30^ .

Vale ressaltar que para estimar o custo do parto pré-termo, em nossa análise foram considerados os custos relativos às primeiras 10 semanas após a data provável do parto e também não foram somados os custos relacionados aos cinco óbitos fetais e dois óbitos neonatais da nossa amostra. Nós consideramos esta uma limitação de nosso estudo. Uma análise de longo prazo poderia trazer resultados ainda mais impactantes quanto ao custo-benefício da implementação do programa de rastreio.

Nosso estudo também apresenta a limitação de não valorar os custos atrelados ao seguimento das mulheres identificadas sob risco de prematuridade. É verdade que, após ser identificado colo uterino curto, cada paciente formará sua percepção de risco e isso pode gerar mais ansiedade, estresse, consultas de pré-natal e perda laboral. Ao mesmo tempo, a construção de uma boa relação médico-paciente, que deve ser premissa de todo atendimento médico e que não agrega custo final, pode trazer os esclarecimentos e orientações necessários para reduzir esse estado de vulnerabilidade e facilitar a adesão ao tratamento proposto ^31^ .

Podemos assim considerar esta análise como um estudo de custo preliminar, um primeiro passo para um olhar mais atencioso à possibilidade de implementar a USTV para medida do colo uterino no segundo trimestre como um programa de rastreio para a prematuridade no Brasil. Entendemos que a decisão de utilizar os custos do tratamento da prematuridade conforme a tabela SUS (subestimados em relação ao custo real) e os valores do tratamento para o colo curto conforme CMED/Anvisa (preços de mercado, maiores que preços de uma possível aquisição direta), em última análise, superestimam os custos de prevenção e subestimam os custos de tratamento e, por conseguinte, subestimam a redução e custos com o rastreamento. Mesmo com essa consideração, encontramos uma redução de custos favorável ao rastreamento.

Entretanto, para o desenvolvimento de resultados mais robustos, será necessária uma análise de custo-efetividade com a modelagem de diversos outros fatores que influenciam diretamente no resultado do custo incremental, como por exemplo, a aceitabilidade à realização do rastreamento, a real adesão ao tratamento oferecido e a perda laboral das mulheres sob maior risco de parto pré-termo, permitindo estimar valores mais condizentes com a realidade através das análises de sensibilidade.

## CONCLUSÃO

Diante do exposto, concluímos que o rastreamento universal do colo curto para a prevenção da prematuridade com USTV no segundo trimestre de gestação, associado à profilaxia com progesterona vaginal para as gestantes identificadas com colo ≤ 25 mm, apresenta menores custos em relação ao não rastreamento dentro de um horizonte temporal de curto prazo, o que sugere uma interessante relação custo-benefício na implementação do rastreio universal do colo curto para a prevenção do parto pré-termo no Brasil. Novos estudos que também considerem a custo-efetividade do tratamento profilático utilizando-se de análises de sensibilidade em diferentes cenários dentro do sistema de saúde brasileiro são necessários para justificar com robustez o rastreamento da prematuridade.
